# Investigation of Quality Enhancement Mechanisms in Tenobe Somen Noodles During Storage and Maturation

**DOI:** 10.3390/foods14183204

**Published:** 2025-09-15

**Authors:** Qifei Wu, Wei Li, Yajing Qi, Shuyi Liu, Zhongwei Chen, Bin Xu

**Affiliations:** School of Food and Biological Engineering, Jiangsu University, Zhenjiang 212013, China; jsdxwqf@163.com (Q.W.); 15751253616@163.com (W.L.); xiaoyataoyao@163.com (Y.Q.); 18252585942@163.com (S.L.); zwchen@ujs.edu.cn (Z.C.)

**Keywords:** hand-stretched noodles, lipid oxidation, protein-lipid interactions, physical properties, storage-induced quality enhancement

## Abstract

Tenobe somen (hand-stretched) noodles are distinguished by their exceptional quality, which is achieved through a unique production method and a characteristic long-term aging process. This aging is closely associated with the oiling and “yaku” procedures. “Yaku” refers to the process of storing dried tenobe somen noodles in a warehouse during the high-temperature and high-humidity rainy season (typically in summer) for a period of time. This process is not merely about storage; rather, it involves complex physicochemical changes in the internal components of the noodles triggered by environmental factors, ultimately endowing the noodles with superior quality. This review systematically examines the critical factors influencing tenobe somen production, including oil selection for anti-adhesion treatment, the evolution of fundamental physicochemical properties, cooking performance, and sensory quality during storage. Particular emphasis is placed on the transformations of lipids, proteins, and starch components, as well as their intermolecular interactions. Recent findings demonstrate that cottonseed oil is especially effective in preventing strand adhesion during processing and contributes substantially to quality enhancement throughout storage. The optimization of noodle quality during aging is largely driven by chemical composition changes and synergistic molecular interactions. Overall, this review provides a comprehensive analysis of the multidimensional mechanisms underlying quality improvement in tenobe somen noodles. The insights gained offer valuable theoretical support for optimizing lipid selection, regulating storage protocols, and promoting the modernization of traditional pasta production technologies.

## 1. Introduction

Tenobe somen (hand-stretched) noodles are a traditional type of handmade wheat noodles that are coated with edible oil on the surface [1]. Compared with ordinary noodles, the production of tenobe somen involves several distinctive steps, including kneading the dough with high-salt water (10–18% salt concentration), applying edible oil, spiral stretching to form a dense gluten network, and the crucial “yaku” storage. During this stage, dried noodles are stored for several months in the hot and humid conditions of the rainy season, typically at 25–30 °C and 85–95% relative humidity [2]. This unique long-term aging process is believed to promote a series of complex biochemical changes within the noodles, such as water migration, lipid oxidation, and gradual modifications of proteins and starches. These transformations ultimately confer the characteristic qualities of tenobe somen, including firm chewiness, low stickiness, and a refreshing taste [3,4]. Owing to these properties, tenobe somen noodles are considered a high-quality product widely appreciated by consumers and are often consumed cold during the summer months. According to the data from the 23rd China Convenience Food Conference, the annual production of tenobe somen noodles in China is approximately 300,000 tons, and demand continues to grow [5]. In general, noodles have a relatively short shelf life and are prone to quality deterioration during long-term storage. For example, dried noodles may soften upon exposure to moisture, while fresh noodles can harden or become sticky after prolonged refrigeration [6]. In contrast, tenobe somen noodles, owing to their distinctive production method and storage process, are able to maintain desirable quality characteristics during extended storage.

The oiling process is a crucial step in the production of tenobe somen noodles, as it prevents noodles from sticking together, improves their overall quality, and their extends shelf life [6]. The choice of oil is of particularly important, since its fatty acid composition, oxidative stability, and viscosity directly influence the effectiveness of the oiling treatment [7]. With respect to fatty acid composition, oils with a higher proportion of saturated fatty acids generally exhibit greater oxidative stability, which is favorable for long-term storage [8]. However, given the biochemical reactions that occur during the storage of the tenobe somen noodles, the oil must also be capable of undergoing moderate oxidation [9]. Furthermore, viscosity plays a key role in ensuring uniform coating and anti-sticking performance. Oils with excessively low viscosity may lead to uneven coating, whereas those with very high viscosity can result in a greasy noodle surface, negatively affecting the eating experience [10]. Therefore, the selection of oils for the oiling process must comprehensively balance the fatty acid composition, oxidative stability, and viscosity.

The “yaku” process, which occurs during the storage stage of tenobe somen noodles, represents another critical step in achieving multidimensional quality development [11]. Under conditions of high temperature and humidity that persist for several months, the moisture content of noodles undergoes dynamic changes, initially absorbing environmental moisture and subsequently reaching equilibrium. These changes significantly influence the noodles’ chewiness and texture. At the same time, cooking properties such as the cooking loss, optimal cooking time, and water absorption capacity also evolve with storage duration. Such transformations are closely related to complex alterations and interactions among lipids, proteins, and starches in the noodles [11]. Starch retrogradation and modifications in the protein network are the primary factors governing the rheological properties of cooked noodles [12]. The degree of starch granule swelling and the physicochemical properties, together with the surrounding protein matrix, collectively determine the final texture and sensory attributes [13]. Notably, lipid oxidation and interactions with proteins play a particularly important role during the “yaku” process, and are regarded as some of the key drivers of the characteristic changes in noodle quality [14]. In addition, alterations in carbohydrates, enzymes, and other minor components during maturation further influence the product quality [15].

Although some studies have examined quality changes during storage, including moisture migration, certain textural parameters, and basic compositional analyses, significant gaps remain in both the mechanistic understanding and systematic research. First, in terms of selecting the optimal anti-sticking oil, most current studies adopt a single-perspective approach, leaving uncertainty about which type of oil best supports the desired “yaku” effect. Second, there is a lack of systematic and continuous monitoring of how key quality indicators evolve throughout the multi-month “yaku” period under different temperature and humidity conditions. As a result, the biochemical events occurring at different “yaku” stages and their contributions to the final noodle quality remain poorly understood. Most importantly, the intrinsic molecular mechanisms through which “yaku” promotes quality improvement are still unclear. For example, the extent to which lipid oxidation products influence protein modifications, thereby enhancing the noodle texture, and the dynamic interactions among starches, proteins, and lipids during maturation remain unresolved.

This review, therefore, focuses on the scientific basis for selecting anti-sticking oils in tenobe somen noodle production. It systematically examines the dynamic changes in multidimensional quality attributes during “yaku” storage and maturation, and it explores the potential biochemical and physicochemical mechanisms responsible for quality improvements. By addressing these gaps, the review aims to advance current understanding and provide insights into both fundamental mechanisms and practical approaches for optimizing tenobe somen production.

## 2. Optimizing Anti-Stick Oil Selection in Tenobe Somen Noodle Production

In the production of tenobe somen noodles, the oiling step is of critical importance. The application of edible oil to the noodle surface serves two primary functions: preventing surface drying and reducing adhesion between strands [2]. The anti-sticking mechanism of oiling is mainly attributed to lubrication and isolation [16]. When a layer of oil is applied between two surfaces, it penetrates the microscopic gaps, fills surface irregularities, and reduces both the contact area and friction between them [17]. As a result, the surfaces slide more easily against each other, lowering the risk of sticking. In addition, the oil layer acts as a barrier that minimizes direct contact between surfaces [18]. This isolating effect prevents adhesion caused by surface roughness, chemical interactions, or other external factors. Beyond its role in preventing adhesion, oiling also contributes to moisture retention in the noodles, which helps maintain quality during storage. Furthermore, the oil coating imparts a smooth, slippery texture when eaten, a sensory characteristic valued by consumers [2].

The effectiveness of the anti-sticking treatment is influenced by several factors, including the oxidative stability and viscosity of the oil, as well as its adhesion to the noodle surface. These properties collectively determine the uniformity of coating, the degree of lubrication, and ultimately the eating quality of the final product.

### 2.1. Stability of Different Oils

Edible oils are primarily composed of triglycerides, which are generally liquid at room temperature [19,20]. Triglycerides are formed by the esterification of one glycerol molecule with three fatty acids [21]. The oxidative stability of oils is mainly determined by two factors: the fatty acid composition and antioxidant content [22]. When the fatty acid composition is similar, the antioxidant content becomes the key factor influencing stability [23]. Saturated fatty acids exhibit greater molecular stability and are less prone to oxidation, whereas unsaturated fatty acids, due to the presence of double bonds in their structure, readily undergo oxidation reactions with oxygen [24]. Naturally occurring antioxidants in oils, such as vitamin E and oryzanol, play an important role in delaying oxidation, protecting oils from the effects of light and oxygen, and maintaining stability [23].

In the Chinese food industry, commonly used edible oils include soybean oil, rice bran oil, and palm oil. In contrast, the production of Japanese tenobe somen noodles typically employs cottonseed oil as the surface-coating medium [2]. Soybean oil, while widely available, contains a high proportion of unsaturated fatty acids, particularly linolenic acid, which makes it highly susceptible to oxidation and rancidity [25]. Research indicates that the oxidation rate of linolenic acid is approximately twice that of linoleic acid and twenty-five times that of oleic acid [26]. Although this oxidative process reduces the free fatty acid content during storage, it can hinder the “yaku” maturation process of tenobe somen noodles. Rice bran oil, by contrast, has a higher proportion of saturated fatty acids and is rich in natural antioxidants such as vitamin E and γ-oryzanol, which provide excellent oxidative stability and contribute to a longer shelf life [27,28]. Palm oil also demonstrates good stability due to its relatively low unsaturated fatty acid content. However, because saturated fatty acids account for nearly 50% of its composition, long-term consumption of palm oil may elevate blood triglyceride and low-density lipoprotein cholesterol levels; thus, it should be used cautiously [26,29].

Cottonseed oil, with its low proportion of unsaturated fatty acids and relatively stable chemical structure, exhibits superior oxidative stability and low reactivity with oxygen. These characteristics make it particularly suitable for coating noodles, as it effectively resists rancidity during storage and helps maintain product quality [12]. Although rice bran oil and palm oil also offer certain stability advantages, cottonseed oil demonstrates a better balance between oxidative stability and long-term nutritional safety. For this reason, it is preferred in tenobe somen noodle production. The fatty acid composition of several edible oils is summarized in Table 1.

In addition to the fatty acid composition, the acid value and peroxide value are important indicators for evaluating oil stability [23]. The acid value reflects the content of free fatty acids generated through triglyceride hydrolysis during storage. Higher acid values indicate more severe triglyceride degradation and poorer oil stability [30]. Free fatty acids also promote free radical formation, accelerating oxidative chain reactions and increasing susceptibility to rancidity [19]. The peroxide value serves as a critical parameter for assessing oxidative rancidity, particularly during thermal processes such as frying [31]. Lipid oxidation initially produces hydroperoxides and peroxides, which subsequently decompose into volatile compounds including fatty acids, alcohols, aldehydes, and ketones [32]. Elevated peroxide values, therefore, indicate greater oxidative degradation and a significant decline in oil stability.

**Table 1:** Fatty acid composition of oils.

Types of Oil	Palmitic Acid (%)	Stearic Acid (%)	Oleic Acid (%)	Linoleic Acid (%)	Linolenic Acid (%)	Other Fatty Acid Components (%)	References
Cottonseed oil	20.80	2.10	16.10	50.70	/	10.30	[33]
Rice bran oil	14.97	1.93	40.85	31.42	1.65	9.18	[34]
Soybean oil	10.67	3.98	22.75	51.45	7.07	4.08	[35]
Palm oil	45.00	3.50	40.50	10.00	/	1.00	[36]
Peanut oil	12.60	5.14	42.24	31.37	0.11	8.54	[37]
Rapeseed oil	4.82	4.91	48.68	17.92	8.67	15.00	[38]
Sesame oil	7.86	5.25	39.10	45.50	0.26	2.03	[39]
Corn oil	13.76	2.21	29.67	51.70	1.02	1.64	[40]
Tea seed oil	11.92	2.95	83.19	0.08	0.45	1.41	[41]
Olive oil	13.50	4.46	72.71	6.07	0.72	2.54	[42]
Sunflower oil	7.97	4.87	22.54	62.18	0.20	2.24	[43]

### 2.2. Viscosity of Different Oils

**Figure 1:**
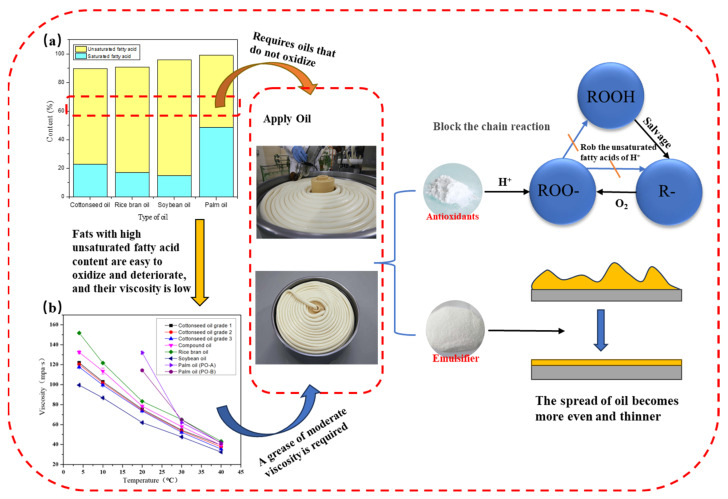
Mechanisms by which food additives improve oils: (**a**) ratio of unsaturated to saturated fatty acids in various oils (data from current laboratory research); (**b**) viscosity levels of different oils (data from current laboratory research).

The viscosity of oils is an important physical parameter that characterizes their properties. Differences in viscosity exist among various oils and fats, and temperature is the main influencing factor [44]. In general, the viscosity of liquids decreases as temperature increases. This occurs because rising temperatures provide liquid molecules with more energy, increase their movement speed, and widen the distance between molecules. As a result, intermolecular forces weaken, molecular mobility increases, and viscosity decreases. Fasina et al. [45] measured the viscosities of 12 vegetable oils under different temperature conditions and found that the viscosity of all oils declined with increasing temperature, showing similar variation trends. A comparable trend is illustrated in Figure 1b, and Table 2 provides the specific viscosity values in the range of 5–40 °C.

In addition, as shown in Figure 1b, the viscosity is also influenced by the fatty acid composition [46]. Saturated fatty acids, with their linear molecular structure and tight packing, typically result in higher viscosity. In contrast, unsaturated fatty acids exhibit lower viscosity because the kinks introduced by double bonds prevent close molecular packing [47]. Yang et al. [48] reported that viscosity is positively correlated with effective the carbon number and oleic acid content but negatively correlated with the polyunsaturated fatty acid content. Bonnet et al. [49] applied the Arrhenius model to establish the relationship between viscosity and temperature in olive oil, and further correlated viscosity with the fatty acid composition using empirical formulas. Their findings confirmed that even at constant temperature, the fatty acid composition significantly affects oil viscosity.

In noodle production, achieving an appropriate viscosity is crucial for ensuring uniform oil film coverage. Cottonseed oil, with its moderate viscosity, can form a stable coating on noodle surfaces. This not only provides an effective anti-sticking effect but also imparts a desirable luster. Such performance is difficult to achieve with oils of higher viscosity (e.g., palm oil) or lower viscosity (e.g., soybean oil).

### 2.3. Oil Adhesion on Noodle Surfaces

As shown in Figure 1, the amount of oil adsorbed on the surface of noodles and the uniformity of its distribution are key factors that influence noodle quality. The adsorption capacity of oil directly reflects the bonding strength between oil molecules and the noodle surface. This determines whether a stable and effective anti-sticking oil film can form, which in turn affects the final taste and flavor characteristics of noodles [48]. If the adsorption capacity is too low, the oil film may not fully cover the noodle surface, resulting in insufficient anti-sticking performance, a dry mouthfeel, and a weak aroma. On the other hand, excessive adsorption can make the surface of noodles overly greasy, negatively impacting the eating experience and consumer acceptance. The uniformity of the oil distribution is equally critical for the overall quality of the product [10,50]. Uneven oil application can lead to localized greasiness or dryness, as well as inconsistent surface gloss and texture, which directly reduce the sensory appeal [51,52].

To scientifically assess the sensory effects of oil adhesion, several evaluation methods can be employed. At the visual level, high-resolution imaging systems can be used to quantitatively analyze the uniformity of oil film coverage and the consistency of light reflection on noodle surfaces under standard lighting conditions [53]. From a tactile perspective, trained evaluation panels can score the smoothness and greasiness [54]. In practical applications, processes such as spraying, dipping, or roller coating are commonly used to achieve a desirable balance between the anti-sticking performance and sensory quality [55,56]. For instance, Nurhayati et al. [56] reported that uniformly coating wet noodles with 5% vegetable oil by spraying or dipping effectively prevented sticking and significantly reduced microbial contamination.

## 3. Quality Changes of Tenobe Somen Noodles During the Aging Period

### 3.1. Changes in the Basic Properties of Tenobe Somen Noodles During Maturation

As storage and ripening progress, the fundamental characteristics of tenobe somen noodles, including the moisture content, elasticity, and breaking stress, change significantly, thereby affecting the overall quality [57,58]. Among these, the moisture content is particularly critical. After production, the moisture content of noodles upon storage is generally slightly above 15%, although it is often lower in practice [59]. Under dry conditions, moisture evaporates rapidly, reducing the content to below 14%. Conversely, in high-humidity environments, the moisture content may increase, even exceeding the initial level [60]. Moisture migration, evaporation, and absorption are influenced not only by ambient temperature and humidity but also by internal structural and compositional changes in the noodles [61,62]. These variations affect other key properties, such as the elasticity and breaking stress.

Elasticity evolves in stages during storage. Two main maturation phases are typically observed. The first “yaku” occurs around the 6th to 8th month of storage, during the hot and humid plum rain season, with temperatures of 20–25 °C and relative humidity levels of 60–70%. The second “yaku” takes place around the 18th to 20th month, during the subsequent rainy season [13]. During these stages, the noodles’ hardness increases, and their elasticity undergoes notable changes. These modifications primarily result from interactions between proteins and lipids, particularly free fatty acids [12]. Free fatty acids bind to gluten proteins, strengthening the gluten network and enhancing the elasticity [63]. However, excessive water loss can impair gluten hydration, reducing elasticity [64]. Therefore, changes in elasticity reflect the combined effects of gluten network development and the moisture content.

Breaking stress, defined as the maximum external force a noodle can withstand before breaking, also changes during maturation [65]. Following the “yaku” process, tenobe somen noodles typically display increased hardness, reduced cohesiveness, and a higher Young’s modulus. These changes indicate structural modifications caused by triglyceride hydrolysis, which releases free fatty acids that affect starch gelatinization and gluten functionality, ultimately altering the noodles’ breaking stress [66]. Additionally, the interaction between a strengthened gluten network and the water content further determines the resistance to breakage. Dry noodles, with insufficient moisture, tend to be brittle and exhibit lower breaking stress, while noodles stored under moderate humidity retain greater toughness.

These property changes are interrelated and collectively determine the final noodle quality. The moisture content is a fundamental factor influencing the dough’s processability and texture. Optimal moisture levels, generally 45–50%, allow for full hydration of gluten proteins, leading to a continuous, uniform, and resilient gluten network that is essential for high elasticity. Excessively high moisture enhances the gluten extensibility but may reduce the breaking stress due to softening, while low moisture prevents proper gluten development, lowering both the elasticity and fracture stress. After the second “yaku”, at approximately 20 months of storage, tenobe somen noodles generally achieve an optimal balance of hardness, elasticity, and water absorption. At this stage, the taste, chewiness, and overall sensory quality reach their peak, making the noodles most suitable for consumption.

### 3.2. Changes in Cooking Properties of Tenobe Somen Noodles During Maturation

During the maturation of tenobe somen noodles, their cooking performance undergoes significant changes, influenced by factors such as the production process and the unique “yaku” phenomenon. The cooking performance is typically evaluated using indicators such as the cooking time, cooking loss, and water absorption rate [67].

The cooking time refers to the duration required for noodles to reach an optimal state for consumption [68]. During the stretching and shaping process of tenobe somen noodles, a loose and porous structure is formed, the gluten network is fully developed, and starch granules are tightly encapsulated [69]. This structure facilitates rapid heat transfer and accelerates starch gelatinization, resulting in shorter cooking times. Katagiri et al. [70] reported that the cooking time of tenobe somen noodles is shorter than that of machine-made noodles. With extended storage, the gluten structure gradually weakens, interactions between starches and lipids decrease, and water absorption accelerates, further shortening the cooking time [9]. The reduction in cooking time reflects changes in the noodle microstructure, which allows rapid cooking and produces a smoother texture. However, it may also cause slight softening and minor loss of structural integrity, reflecting a balance between traditional processing and the noodle structure.

Cooking loss represents the substances dissolved in water during cooking, including starches and proteins. During storage, transformations in proteins, lipids, and starches during the “yaku” period affect the amount and composition of dissolved substances, influencing both the nutritional value and flavor [12]. Cooking loss is also affected by the noodle composition and processing methods [16,71,72,73]. Zang et al. [74] found that dough with higher moisture and gluten contents (>30%) exhibited lower dry matter loss during steaming. This is attributed to glutenin forming an elastic network through disulfide bonds and gliadin providing viscous support; an optimized ratio of approximately 1:1 enhances the gluten network’s ability to encapsulate starch granules, reducing dissolution. Similarly, Zhang et al. [75] reported that cooking loss correlates with the noodle moisture content and soaking conditions. The water absorption rate measures the ability of noodles to absorb water during cooking and is calculated from the weight change before and after cooking. Changes in water absorption are related to gluten degradation and lipid oxidation [76]. It is generally believed that after the first “yaku”, the water absorption rate changes smoothly and reaches the optimal state. Niihara et al. [59] observed that water absorption decreases during the “yaku” period as gluten proteins and lipids transform over storage time. Moreover, water absorption is negatively correlated with the optimal cooking time. Hatcher et al. [77]. demonstrated that noodles made from different Canadian wheat flours with higher water absorption exhibited shorter optimal cooking times.

In summary, the cooking quality of tenobe somen noodles is affected by multiple factors, with the gluten structure, lipid oxidation, and starch–protein interactions playing dominant roles during the maturation and “yaku” processes.

### 3.3. Changes in the Edible Quality of Tenobe Somen Noodles During Maturation

The edible quality of tenobe somen noodles evolves through a complex physicochemical process during storage and maturation, driven by interactions among proteins, starches, and lipids [13]. Changes in texture characteristics, including the hardness, elasticity, smoothness, and chewiness, reflect transformations in internal components and directly influence consumers’ sensory experiences and acceptance [78].

Hardness changes during the aging process are closely associated with the “yaku” phenomenon. After the first “yaku” stage, the noodle hardness increases significantly, often perceived as desirable chewiness in consumption. This moderate increase aligns with consumer expectations for the texture and toughness of high-quality noodles. During the second “yaku” stage, the hardness does not increase excessively, preventing negative perceptions such as overly hard or difficult-to-chew noodles [59]. Hardness changes are also closely linked to the moisture content [63]. Reduced moisture generally increases hardness, whereas appropriate moisture retention maintains the flexible texture preferred by consumers [79].

Elasticity is influenced primarily by protein–lipid interactions. Niihara et al. [11] observed that elasticity changes occur alongside increases in hardness when studying gluten protein and fatty acid interactions [59]. Gluten proteins in tenobe somen noodles, composed mainly of gliadin and glutenin, form a network structure in the presence of water, imparting elasticity and toughness [80,81,82]. During maturation, prolonged storage promotes the formation of disulfide bonds and cross-linking within the gluten network, strengthening interactions between protein molecules and increasing the noodle toughness [83]. Noodles with moderate elasticity display good extensibility and soft resistance during chewing, producing positive sensory evaluations. However, excessive elasticity may result in stiffness and reduced consumer acceptance. Therefore, the specific relationship between elasticity and consumer acceptance still needs to be clarified through systematic sensory experiments.

The smoothness and chewiness also improve during the “yaku” process. Research shows that matured tenobe somen noodles develop a crisp and clean taste, reflecting enhanced smoothness and chewiness [12]. This improvement is attributed to interactions between free fatty acids and gluten proteins during storage, particularly under the high-temperature, high-humidity conditions of the rainy season [84]. These protein–lipid interactions modify the properties of gluten proteins, enhancing both the texture and overall taste of the noodles.

Overall, the evolution of edible quality in tenobe somen noodles is driven by changes in proteins, starches, and lipids, as well as interactions between lipids and proteins and between lipids and starches. These molecular transformations underlie the characteristic texture, chewiness, and sensory attributes that define high-quality tenobe somen noodles.

## 4. Chemical Changes in Tenobe Somen Noodles During the Maturation Process

### 4.1. Lipids

**Figure 2:**
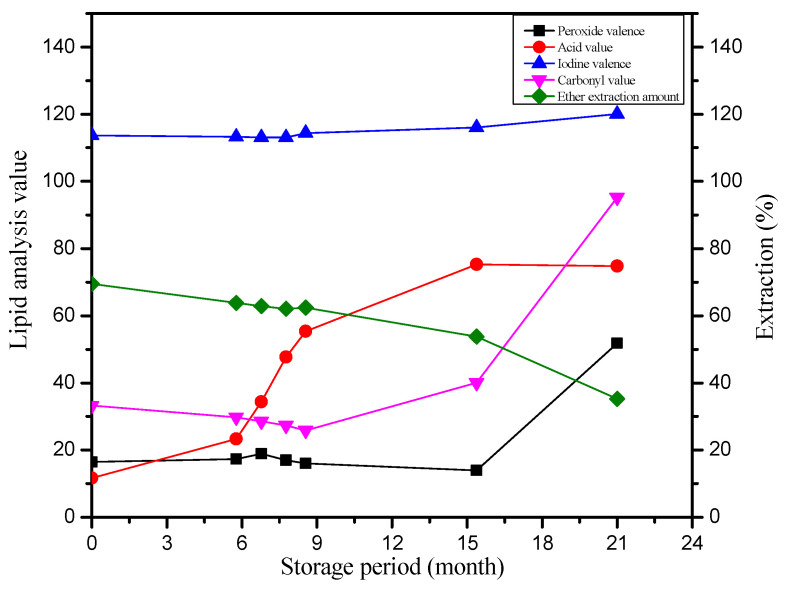
Lipid changes during the storage of tenobe somen noodles. Open Access permission reprinted and adapted from Ritsuko et al. [15,59].

The lipids in tenobe somen noodles undergo distinct changes during the two “yaku” periods. During the first “yaku” period, lipids remain relatively stable, with minimal oxidation. Significant lipid oxidation is observed only during the second “yaku” period [15]. As shown in Figure 2, the acid value of lipids increases markedly during storage, particularly in the first “yaku” period, rising from an initial 10 mg KOH/g to 27 mg KOH/g. This increase indicates ongoing lipid hydrolysis [85]. However, the acid value does not continue to rise in the second “yaku” period. This phenomenon may result from changes in lipase activity [86]. While the high temperature and humidity of the first “yaku” period can activate lipase and promote hydrolysis, prolonged high temperatures can denature the enzyme, damaging active sites such as serine residues. As a result, the lipase activity declines significantly during the second “yaku” period [12,87]. In addition, lipid oxidation produces hydroperoxides and aldehydes, which can bind to lipase active sites to form irreversible complexes, further inhibiting lipid hydrolysis [88].

Lipid oxidation can be monitored using indicators such as the iodine value, peroxide value, and carbonyl value [59,89]. Niihara et al. [59] studied lipids extracted with ether during different storage periods, including both “yaku” stages. During the first “yaku” period, the iodine values remained stable at 110–120 g I_2_/100 g, indicating minimal oxidation of unsaturated fatty acids. The peroxide value stayed below 16 meq/kg, and the carbonyl values remained low at 1.1–1.3 nmol/mg, reflecting inhibited overall oxidation. In the second “yaku” period, the iodine value gradually increased, indicating oxidation of unsaturated fatty acids. The peroxide values rose rapidly to 4.8–5.2 meq/kg, marking the onset of lipid oxidation, while the carbonyl values increased to 3.9–4.2 nmol/mg, reflecting progression to the middle and late stages of oxidation. These results indicate that lipid oxidation is largely controlled before the second “yaku” period and becomes pronounced only during this stage. The specific changes are shown in Figure 2 and Table 3.

Studies on the fatty acid composition during aging revealed minimal changes, although the proportion of linoleic acid increased slightly [59]. This increase may result from the high linoleic acid content in wheat flour lipids [90]. During storage, the cottonseed oil coating the noodle surfaces gradually becomes difficult to extract, while internal wheat lipids remain stable, resulting in a relative increase in linoleic acid in extracts. Surface lipid oxidation products can react with proteins and become insoluble, but oxidation does not readily penetrate the internal lipids, allowing them to remain stable for extended periods. This contributes to overall lipid stability. The stability of lipids during storage is also influenced by the moisture content [91,92]. Niihara et al. [59] found that noodles stored under high-humidity conditions exhibited minimal reductions in unsaturated fatty acids compared with low-humidity storage, highlighting the protective effect of moisture on lipid stability.

### 4.2. Proteins

**Figure 3:**
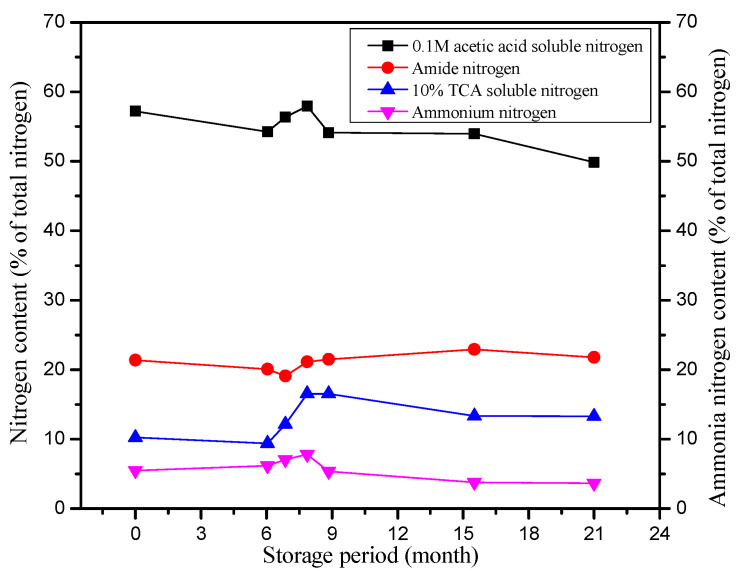
Protein changes during the maturation of tenobe somen noodles. Open Access permission reprinted and adapted from Ritsuko et al. [15].

During the maturation of tenobe somen noodles, proteins undergo changes in two distinct stages—subtle modifications during the first “yaku” period and pronounced structural transformations during the second “yaku” period, as illustrated in Table 3 and Figure 3. In the early stage, when lipid oxidation is minimal, proteins experience minor conformational and hydrolytic adjustments without significant covalent bond formation or cleavage.

During the first “yaku”, wet gluten loses its fluidity, and its water absorption capacity decreases [85]. Niihara et al. [59] reported that using a farinograph to test plain flour powder, protein water absorption decreased by 5–8% compared with the initial state. The high temperature and humidity enhance the protease activity, initiating protein hydrolysis. Consequently, TCA-soluble nitrogen increased by approximately 20%, while free amino acids rose by 10%. These products were primarily small molecules generated through hydrolysis, with no subsequent covalent reactions [93,94]. Upon entering the second “yaku” period, lipid oxidation becomes significant, triggering major structural changes in proteins. Covalent reactions and cross-linking dominate this stage. Gluten proteins form high-molecular-weight polymers through disulfide bonds (-S-S-) and Schiff base (C–N) reactions, induced by lipid oxidation products. This covalent polymerization results in a notable decrease in protein solubility [15]. As shown in Figure 3, the soluble nitrogen in 0.1 M acetic acid declined to 15%, representing a 16.7% decrease compared to the end of the first “yaku”. In a 6 M urea containing 0.6% mercaptoethanol, the protein solubility decreased to 70%, confirming that covalently cross-linked polymers are difficult to dissolve.

Amino acid analysis indicates significant covalent loss and peptide recombination. All amino acids decreased, with lysine and histidine showing the largest reductions. Small peptides and free amino acids produced during the first “yaku” were incorporated into macromolecular protein polymers via covalent bonds, such as amide linkages. This integration caused 20–30% reductions in TCA-soluble nitrogen compared to the end of the first “yaku”, demonstrating the covalent incorporation of hydrolysis products [95]. Protein gel electrophoresis further illustrates these changes. After the first “yaku”, electrophoretic patterns showed no significant alterations. However, after the second “yaku”, the gray values of major protein bands, such as 30 kDa (gliadin) and 60 kDa (glutenin), decreased, and pronounced tailing appeared above 100 kDa. These observations indicate a reduction in small molecular proteins and the formation of large covalent polymers, confirming substantial structural transformation of proteins [96].

### 4.3. Starch Granule Swelling

**Figure 4:**
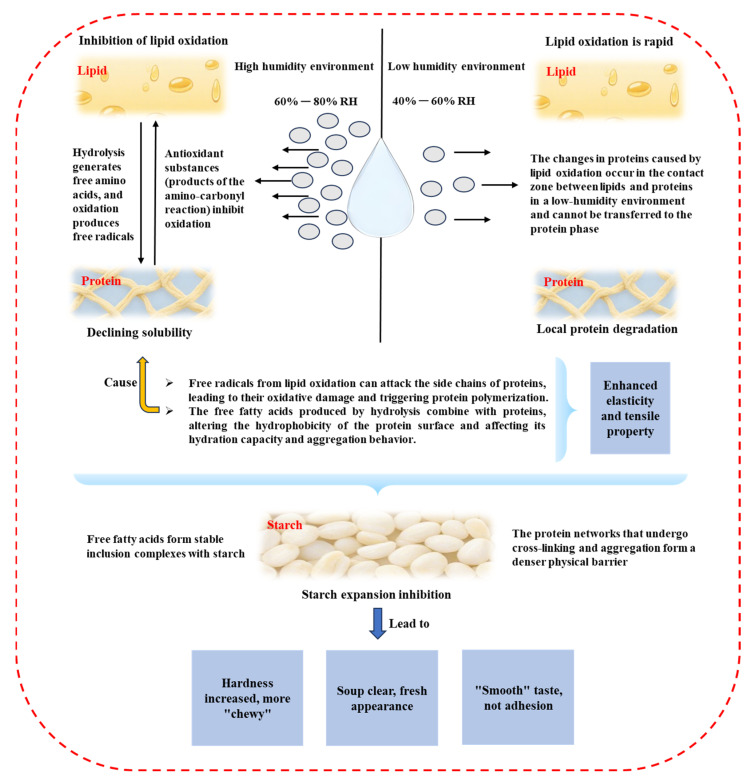
Interaction and quality effects of lipids, proteins, and starches mediated by humidity.

The “yaku” phenomenon during the aging period of tenobe somen noodles significantly influences the swelling properties of starches. Studies have shown that starches from tenobe somen noodles subjected to the “yaku” process exhibit markedly lower swelling compared with untreated plain noodles [66]. After storage during the plum rain season, the swelling capacity of cooked noodles decreases, primarily due to the accumulation of free fatty acids, which inhibit starch swelling [66]. Free fatty acids interact with starch molecules, forming complexes with amylose. These complexes exhibit high thermal stability and low solubility in water, thereby altering the swelling behavior of starch [33,97]. Additionally, fatty acids compete for water, reducing the water’s availability for starch granules and further limiting their swelling. During gelatinization, the presence of fatty acids restricts the unwinding of starch molecular structures, preventing the complete opening of amylose and amylopectin double-helix structures [98,99]. The complexes formed by fatty acids also enhance the thermal stability of starch granules, increasing the gelatinization temperature while reducing the extent of granule swelling [33,85]. Proteins also play a regulatory role in starch swelling [66]. Niihara et al. [66] reported that the addition of bovine serum albumin or α-globulin to wheat starch in salt water significantly reduced starch swelling. Conversely, the low thermal stability of amylase in plain noodles means that changes in amylase activity have a minimal impact on starch swelling during cooking [13,100], as shown in Table 3 and Figure 4.

## 5. Molecular Interactions Under Maturation Conditions in a Simulated System

### 5.1. Lipid Stability and Moisture

In the production and study of tenobe somen noodles, the lipid stability is closely influenced by the moisture content. Moisture is a critical factor that allows gluten to exert its antioxidant effects, thereby modulating lipid oxidation in noodles [101]. As shown in Figure 4, under high-humidity conditions, gluten effectively inhibits lipid oxidation, whereas under dry conditions, lipids in tenobe somen noodles oxidize rapidly [85,102]. Model experiments using a cottonseed oil and wheat gluten mixture demonstrated that increases in lipid peroxide values were significantly suppressed under high humidity. However, this inhibitory effect disappeared under low-humidity conditions [11].

During the maturation of tenobe somen noodles, low-molecular-weight nitrogen-containing compounds, such as amino acids and peptides, are released. These compounds can react with lipid oxidation products, contributing to lipid stabilization. This reaction is also moisture-dependent. For example, experiments conducted by Niihara et al. [11] showed that when cottonseed oil fatty acids were reacted with gluten protein or glycine at 60 °C and 75% relative humidity, a pronounced browning phenomenon occurred in the glycine mixture, accompanied by significant antioxidant effects.

### 5.2. Protein Changes and Moisture in the Gluten–Lipid System

The relationship between protein changes and the moisture content is complex and tightly interconnected, significantly influencing the dynamic states of protein transformation.

During the first “yaku” period in tenobe somen noodle production, lipids remain relatively stable, and protein modifications are minimal. However, in the second “yaku” period, as lipid oxidation progresses, proteins undergo substantial changes [11,103]. Water acts as a medium facilitating interactions between the lipid and protein phases, transporting lipid oxidation products to the protein phase [104,105]. Carbonyl compounds generated by lipid oxidation react with amino groups, causing notable reductions in lysine and histidine residues and thereby altering the protein properties. At the same time, antioxidant compounds from the protein phase can migrate to the lipid interface via water, exerting protective effects against oxidation, as shown in Figure 4.

In contrast, under dry conditions, protein changes exhibit distinct patterns [106]. Overall the protein modifications are limited but the degradation of sulfur-containing amino acids such as cysteine and methionine is more pronounced compared to high-humidity environments. This is because sulfur-containing amino acids are highly sensitive to free radical reactions, with radicals generated in the lipid phase acting as the primary reactive species [107]. Furthermore, the increase in 10% trichloroacetic acid (TCA)-soluble nitrogen is more significant in dry conditions than under high humidity. However, this increase results from localized reactions at the protein–lipid interface rather than from global protein degradation [102,108,109].

### 5.3. The Impact of Sugar Reduction on Tenobe Somen Noodles

During the “yaku” period of tenobe somen noodles, reducing sugars can participate in initiating chemical changes in proteins [15,93]. Reducing sugars, such as glucose and arabinose, can undergo Maillard reactions with amino groups in proteins under high-temperature and high-humidity conditions, producing browning compounds, including melanoidin-like substances. These browning products exhibit antioxidant properties and can delay lipid oxidation [110].

Regarding interactions between wheat gluten and reducing sugars, different sugar types have distinct effects on gluten properties. Ritsuko et al. [15] demonstrated that under high-temperature and high-humidity storage conditions, hexoses (e.g., glucose) had minimal influence on the state of wet gluten, whereas pentoses (e.g., arabinose) showed more pronounced effects. Further studies indicated that arabinose, a pentose, had a significantly greater ability than glucose, a hexose, to alter the physical properties of gluten [15,83]. Thus, while both hexoses and pentoses interact with gluten, pentoses are more likely to modify gluten’s physical characteristics under high-temperature and high-humidity conditions.

However, analyses of reducing sugar contents in tenobe somen noodles show that monosaccharides account for approximately 0.5% of the noodles, with 80–90% being glucose, with only trace amounts of pentoses [93]. Ritsuko et al. [15] supplemented the water extract of somen noodles with pentoses at levels equivalent to those naturally present and observed negligible impacts on product quality during storage. Therefore, to enhance the quality of hand-rolled noodles, a small amount of pentose may be added during the noodle-making process. However, it should be noted that excessive addition may result in over-browning or abnormal flavor. The optimal concentration requires further research and should be carefully balanced with the protein and lipid oxidation systems to avoid negative effects.

## 6. Conclusions and Future Prospects

The quality improvement of tenobe somen noodles during storage and aging is influenced by multiple factors, including the selection of anti-adhesion oils, physico-chemical changes, and molecular interactions. Studies have demonstrated that the physicochemical properties of anti-adhesion oils play a decisive role in the long-term storage stability of tenobe somen noodles. Cottonseed oil shows notable advantages in delaying lipid deterioration due to its low unsaturated fatty acid content and high oxidative stability. However, its comprehensive applicability still requires systematic validation through adsorption kinetics and interfacial viscosity characteristics.

During storage, tenobe somen noodles exhibit characteristic “yaku” phase evolution. The first “yaku” phase is dominated by lipid hydrolysis and mild protein depolymerization, whereas the second “yaku” phase is characterized by free radical-mediated lipid peroxidation and reduced protein solubility, resulting in an increased crosslinking density within gluten networks and restricted starch granule swelling. Moisture plays a key regulatory role in these processes; high humidity (RH > 65%) suppresses lipid oxidation while promoting amino-carbonyl reactions, whereas low humidity accelerates lipid oxidative damage through enhanced free radical chain reactions. The current research mainly focuses on single-component changes, lacking comprehensive analyses of multi-factor synergistic effects, such as interactions among lipids, proteins, and starches, as well as the influence of oil-coating gradients on noodle quality. Future studies should develop accelerated storage models to replicate authentic “yaku” phase conditions; systematically track multi-stage indicators including water migration, elasticity, and cooking loss; and integrate molecular interaction analyses to reveal phase-specific regulatory mechanisms. It is not only expected to deeply explain the scientific principle behind the “yaku” phenomenon but to also provide a theoretical basis for the modernization of traditional flour food technology and quality improvement, and to provide a useful reference for the storage and quality control of high-moisture food.

## Figures and Tables

**Table 2:** Dynamic viscosity levels of oils at different temperatures (mPa·s).

Types of Oil	5 °C	10 °C	20 °C	30 °C	40 °C
Cottonseed oil grade 1	122 ± 1.25 c	103 ± 0.35 c	75.4 ± 0.35 e	54.4 ± 0.92 d	39.4 ± 0.35 c
Cottonseed oil grade 2	120.4 ± 0.72 c	101.7 ± 0.31 cd	74.3 ± 0.12 ef	53.5 ± 0.12 e	37.7 ± 0.12 d
Cottonseed oil grade 3	117.7 ± 0.42 d	99.6 ± 0.6 d	73.6 ± 0.35 f	52.2 ± 0.6 f	35.4 ± 0.2 e
Compound oil	132.5 ± 1.5 b	113.6 ± 3.0 b	78.4 ± 0.35 d	58 ± 0.35 c	39.2 ± 0.35 c
Rice bran oil	152 ± 0.7 a	121.8 ± 0.87 a	83.3 ± 0.58 c	65 ± 0.35 a	43.2 ± 0.6 a
Soybean oil	99.6 ± 0.6 e	86.8 ± 0.35 e	62 ± 0.35 g	47.6 ± 0.35 g	32.4 ± 0.2 f
Palm oil (PO-A)	/	/	132 ± 1.2 a	62 ± 0.35 b	41.8 ± 0.35 b
Palm oil (PO-B)	/	/	114.4 ± 0.92 b	64.8 ± 0.2 a	41.4 ± 0.6 b

Different lowercase letters in the same column indicate significant differences (*p* < 0.05). The data are sourced from laboratory research.

**Table 3:** The changes in the main components of tenobe somen noodles and the formation of product texture during different “yaku” periods.

Ingredients	Biochemical Indicators (Units)	After the First “Yaku” (Stored for 6 to 8 Months)	After the Second “Yaku” (Stored for 18 to 20 Months)	Product Texture	References
Lipids	Acid value (mg KOH/g)	Significant increase (Increase from 10 meq/kg to 25–30 meq/kg)	Basically remain stable	Free fatty acids generated from hydrolysis form insoluble complexes with amylose, significantly inhibiting the gelatinization degree and swelling power of starch granules. This directly leads to increased hardness and reduced stickiness of the cooked noodles, resulting in a smoother mouthfeel.	[12]
	Peroxide value (meq/kg)	Remain unchanged (16–17 meq/kg)	Significant increase (25–30 meq/kg)	Volatile substances such as carbonyl compounds generated by lipid oxidation participate in the Maillard reaction, creating a unique umami flavor.	[13]
	Ether extract (%)	Slow reduction	Significant reduction (down to initial 50%)	Lipids become inextractable due to hydrolysis and oxidation reactions, resulting in a dry appearance.	[70]
Proteins	Solubility in 0.1 M acetic acid (%)	No significant difference (maintained at 58–60%)	Significantly reduced	Proteins undergo intermolecular cross-linking and polymerization, forming a more compact and robust three-dimensional network structure, which enhances the hardness and elastic modulus of noodles, and provides excellent chewiness and tensile strength.	[59]
	Wet gluten yield	Hard to group, loss of liquidity	Almost impossible to extract	The protein undergoes irreversible changes and its water absorption rate decreases.	[66]
Starch	Swelling power after cooking	Starch granule swelling inhibition	Further swelling suppression	The expansion of starch particles is restricted, making the noodles more chewy.	[33]
	Solubility after cooking (%)	Slightly down(from 17.6% to 15.0%)	Further decline	The dissolution of amylose is reduced, the cooking loss is decreased, and the noodle soup is clearer.	[66]

## Data Availability

No new data were created or analyzed in this study. Data sharing is not applicable to this article.
